# A qualitative investigation of perceptions towards antibiotics by members of the public after choosing to pledge as an Antibiotic Guardian

**DOI:** 10.1111/hex.13677

**Published:** 2022-11-23

**Authors:** Lorna Flintham, Diane Ashiru‐Oredope, Jordan Charlesworth, Roger Harrison, Elizabeth Dalgarno

**Affiliations:** ^1^ Epidemiology and Public Health Group, School of Health Sciences, Faculty of Biology, Medicine and Health The University of Manchester Manchester UK; ^2^ HCAI, Fungal, AMR, AMU & Sepsis Division, UK Health Security Agency London UK

**Keywords:** Antibiotic Guardian, antibiotic resistance, behavioural change, education, engagement

## Abstract

**Introduction:**

Antimicrobial resistance is one of the biggest threats facing global humanity. In 2014, Public Health England (now the UK Health Security Agency) launched the Antibiotic Guardian (AG) campaign as a national health promotion initiative to increase public and health professionals' commitment to reducing the threat of antibiotic resistance (ABR). The aim of this research study was to gain a snapshot of public AG attitudes towards antibiotic use, the AG campaign and illness postpledge.

**Methodology:**

This research used an exploratory study design using thematic and framework analysis of semistructured, in‐depth interviews. A purposive convenience sampling strategy was used to recruit 10 participants; adults in the general population who had registered with and chosen an AG pledge via the AG online platform during November 2020 were eligible for inclusion. Interviews were conducted via Zoom.

**Results:**

Six main themes were identified: campaign awareness, motivators to pledge (uncertainty about the future of ABR, personal gratification, personal responsibility, moral obligation and COVID‐19), perceptions of personal responsibility (and patient perspectives of moral obligation in clinicians), the impact of the campaign and campaign promotion. Pledging appeared to solidify existing perceptions AGs held. Behavioural motivations for responsible antibiotic behaviours stemmed from perceptions of personal responsibility, moral obligation and concerns about ABR. AGs attributed responsibility to variable patterns in overprescribing. Perceptions towards COVID‐19, coinciding with the previously established study period, appeared mixed. AGs were keen to promote responsible perceptions in relation to antibiotics, resistance and the AG campaign. However, poor social acceptability of ABR concern was raised as a barrier to campaign promotion.

**Discussion:**

The AGs' longstanding commitment to antimicrobial resistance demonstrates the importance of a pre‐existing interest in the public's self‐reported judicious behaviours and decision to pledge to an ABR‐focused campaign. Presenting the local and global threat to human mortality and morbidity in a more relatable format in public messaging should be considered in future strategies promoting ABR awareness and shifts in public perceptions. More frequent messaging to existing AGs is further recommended to propagate positive behaviour change among a wider audience.

**Patient or Public Contribution:**

This study was based on interviews with adult members of the public who had pledged to be AGs via the website www.AntibioticGuardian.com. Interviews were based on the public's perceptions of the AG campaign, antibiotic use and ABR.

## 1 INTRODUCTION

Since their wide‐scale introduction in the 1940s, antimicrobials have remained the most effective and widely used drugs for the prevention and treatment of many bacterial, viral and fungal infections in people, animals and the environment. Yet, the growing presence of antimicrobial resistance, in which pathogens naturally develop resistance to the therapeutic effect of antimicrobials, is now the biggest risk to the survival of humans and nonhuman animals. The most recent, comprehensive study estimated that, in 2019, 4.95 million (3.62–6.57) deaths were *associated* with bacterial antimicrobial resistance, of which 1.27 million (95% UI 0.911–1.71) were *attributable* to bacterial antimicrobial resistance.^1^ The research findings support previous estimates predicting 10 million annual deaths worldwide by 2050.^2^ The COVID‐19 pandemic may have exacerbated this risk, with as many as 70% of COVID‐19 patients initially receiving antibiotics, despite a lack of clinical indication.^3^

In 2021, the World Health Organization (WHO), the Food and Agriculture Organization of the United Nations (FAO), the World Organization for Animal Health (OIE) and the United Nations Environmental Programme (UNEP) established a united strategy, the Tripartite Plus, to reduce the magnitude of antimicrobial resistance in terms of associated and attributable deaths.^4^ This follows Sustainable Development Goal objectives set in 2020 to reduce bloodstream infections associated with antimicrobial resistance and earlier initiatives established by the WHO Global Action Plan in 2015.^5^, ^6^ Despite such targets, around 30% of antibiotic prescriptions in outpatient settings have been deemed unnecessary on clinical basis.^7^ In UK Primary care alone, approximately two million NHS antibiotic prescriptions are distributed a month,^8^ of which 20% were thought inappropriate.^9^ When also incorporating inappropriate antibiotic selection, duration and dosage, the total proportion of unnecessary antibiotic use has been estimated at a staggering 50%.^10^

Undoubtedly, COVID‐19 has had a differential impact on the use of antibiotics around the world and placed selective pressures (or lack thereof) on antibiotic resistance (ABR). Reductions in bacterial infections were likely to follow through country‐level ‘lockdown’ measures, major restrictions in travel and public‐focused infection control measures.^11^ Comparatively, misunderstanding of COVID‐19 and poor antimicrobial stewardship resulted in considerable use of antibiotics, often without any clinical need.^12^ A meta‐analysis of data derived from Asia found that broad‐spectrum empirical antimicrobial prescriptions were frequent and 72% of COVID‐19 patients received antimicrobial therapy despite less than 10%, on average, having a fungal or bacterial coinfection.^13^ To complement this known, quantitative evidence of selective pressure on ABR development, this study has qualitatively sourced public viewpoints to add to the field's limited exploration of public perceptions of antibiotic use in relation to COVID‐19.

The initial WHO's Global Action Plan on Antimicrobial Resistance includes five objectives to reduce the risk from antimicrobial resistance, of which Object One made a clear mandate to ‘Improve awareness and understanding of antimicrobial resistance through effective communication, education and training’.^6^ To support the UK's 5‐year antimicrobial resistance strategy, Public Health England (PHE, now the UK Health Security Agency [UKHSA]) established the national AG campaign in 2014. With the ambition of reducing the unnecessary use and demand for antimicrobials, the campaign was developed, in part, to involve, educate and engage the public. Like many public health interventions, the involvement of the general public and patients in identifying, developing and implementing interventions is vital for subsequent effectiveness.^14^ The campaign seeks to increase public and health professional commitment to reducing ABR and provide prospective patients with the motivation required for subsequent education.^15^ This study sought to appraise public commitment by gaining a small cohort's individual perspectives.

**Figure 1:**
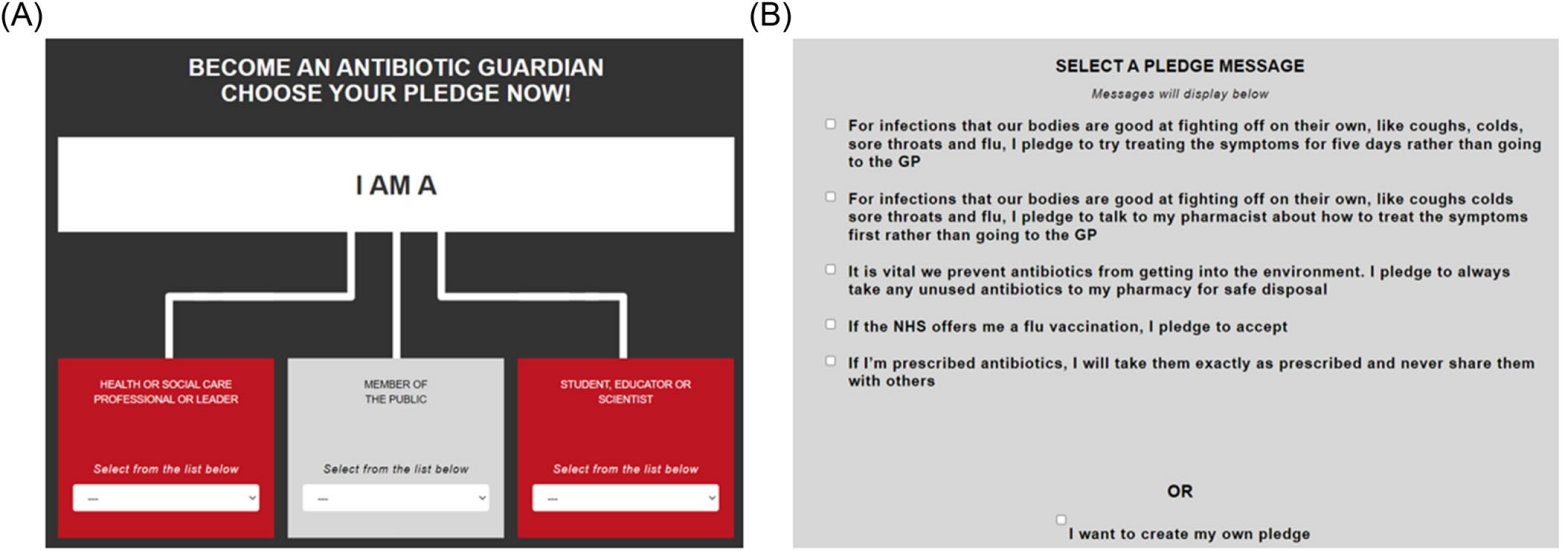
(A) The AG website identity options at the point of pledge. AGs can select whether they are a ‘Health or Social Care Professional or Leader’, ‘Member of the Public’ or ‘Student, Educator or Scientist’. (B) Pledge options for an Adult under the Member of the Public banner.^18^

The campaign focuses on gaining commitment from those accessing it and reducing the risks of antimicrobial resistance by encouraging appropriate health behaviours, especially when seeking clinical input and subsequent use of antibiotics. Anyone with internet access can use this global campaign and pledge to become a ‘guardian’ of antibiotics. The whole campaign is underpinned by evidence of behavioural change and reflects positive approaches of similar strategies open to the public.^16^, ^17^ The online AG campaign collects basic information on the person's background (coded as a ‘member of the public’, ‘health or social care professional or leader’ or ‘student, educator or scientist’). The individual may select one of the available tailored pledges, as shown in Figure 1.

Some similarities with the AG Campaign are shared with previous approaches to public engagement with regard to ABR in the United Kingdom. Notably, the national ‘Andybiotic’ and ‘Keep Antibiotics Working’.^19^, ^20^ However, direct engagement was missing and such approaches focused on more passive health education/awareness raising. More formal, systematic evidence on the value of public awareness interventions, such as 1 including 19 interventions, identified that campaigns trigger a significant effect on public knowledge and antimicrobial stewardship behaviours.^21^ Only one previous study has qualitatively examined AG perceptions in campaign exposure, motivations to pledge and impact of the campaign.^15^ Through semistructured interviews, the researchers found that public and health professional AGs were motivated to pledge due to personal or professional concerns for ABR, but most could not recall their specific pledge.^15^ There remains a dearth of valuable evidence in terms of the distinction between public and health professional perspectives and the potential influence of COVID‐19. This study intends to build on the work of Kesten et al.^15^ by exploring public AG perceptions in isolation while appraising the potential influence of the COVID‐19 pandemic.

### 1.1 Aims/objectives

The aim of this research study was to gain a snapshot of public AG attitudes towards antibiotic use following a pledge made during the month of World Antimicrobial Awareness Week (November) 2020 with specific objectives to explore (1) campaign and antimicrobial resistance awareness; (2) perceptions towards illness, antibiotic treatment and COVID‐19.

## 2 MATERIALS AND METHODS

### 2.1 Eligibility criteria

Participants were eligible for inclusion if they were aged 18 years or over, consented to participate, selected ‘Member of the public’ at the point of pledge, had provided informed consent to be contacted by PHE in the future, were UK‐based and were able to use Zoom. Only those who made their pledge in November 2020, the month including World Antimicrobial Awareness Week, were selected.^22^

### 2.2 Sampling strategy and data collection

A purposive convenience sampling strategy was used with a gender and age balance similar to the main AG cohort.^23^ AG demographic information, such as age, gender and ethnicity, are not collected as part of the current campaign strategy; therefore, it was not possible to stratify the contacted AG sample.

### 2.3 Recruitment

A recruitment email was sent to all eligible AGs, as identified from the central database for the AG campaign, on the 15th June 2021 with a further follow‐up 2 weeks later. The recruitment email presented the participant information sheet, a written consent form and a link to an online survey (Qualtrics.co.uk). The survey sought the participant's first name, gender, age band (18–20, 21–30, 31–40 etc.), ethnicity and email address for further contact. Interview slots were offered between 8 AM and 8 PM 7 days a week to avoid selection bias associated with only allowing individuals with certain working hours or shift patterns to participate.

### 2.4 Ethical considerations

A timetable of available interview times was provided; participants were asked to select slots that would be convenient for the Zoom interview. All participants were informed of how their data may be used before the interview started and all were reassured of their right to withdraw their data from the project at any point before a specified date (31 July 2021).

### 2.5 Participant interviews

All participant interviews took place across June and July 2021, 7–8 months after their pledge. Given the diverse geographic location of AGs and COVID‐19 restrictions present at the time of data collection, interviews were conducted via the web‐based Zoom platform. Participants chose whether their camera was on or off during the interview depending on their personal preference and device camera capabilities. This choice, alongside allowing participants to reside in a comfortable and familiar environment, may have led to more forthcoming responses.^24^ A topic guide was developed after a review of the study published by Kesten et al.,^15^ the only other published qualitative study examining the campaign, and questions were assessed by leaders of the campaign and qualitative research experts for content, purpose, design and relevance. The topic guide was used to direct the semistructured interviews towards relevant areas of questioning: pledging to the campaign, prescribing, antibiotic use and propagating campaign awareness.

### 2.6 Data analysis

Data surrounding AG pledges were analysed according to previously published methodology.^25^ One researcher (L. F.) recorded and transcribed all interviews before analysis. The transcription function within the Zoom software was used to generate a transcript; this transcript was later checked for accuracy and recordings were deleted immediately after transcription to comply with research governance. Framework analysis was used to analyse the transcribed interview data. Framework analysis initially involved familiarization with the data, by re‐examining the recorded interviews and rereading through the corrected transcripts until the researcher was familiar with them in their entirety.^26^

In the second step of framework analysis, thematic analysis was conducted by L. F. to develop a coding scheme. The thematic analysis involved analysing the data to identify common or recurrent themes in the participants' responses.^26^ This was a comparative process by which different participants' responses were gathered and compared. A ‘scissors and paste’ approach was taken and coding schemes were developed to identify core ideas. Themes in the data were identified and these became labels for the codes. Next, as per framework analysis guidance,^26^ codes were applied to the full data set in a process of indexing. In the fourth step, charting, the data was rearranged by thematic content. Charts were developed to contain summaries of the data, by theme, with the range of interview data systematically documented under each theme. This was useful for comparing data across codes and allowing the whole range of phenomena to be observed while examining relationships between codes.^26^ While themes were discussed and refined with supervisor E. D., as this was conducted as a Master's dissertation, the coding, development of themes and analysis was conducted by L. F.

## 3 RESULTS

The number of public adults who pledged to be an AG in November 2020 and agreed to further contact was 135, of whom all were emailed the information about the study and participation. The pool consisted of 102 members of the public who pledged as an ‘Adult’, 25 ‘Family Members’, 7 ‘Pet/Horse Owners’ and 1 ‘Farmer’.

Of the 135 AGs who were contacted, 14 gave consent, of whom 2 did not respond to emails from L. F. and 2 respondents did not provide a correct email address. The remaining 10 participants were interviewed via Zoom. The sample size is representative of all public individuals who pledged during the most recent month of World Antibiotic Awareness Week (and during the height of the COVID‐19 pandemic) who were willing and able to participate in the research. Likewise, smaller sample sizes promote a closer association between the researcher and respondents, increasing the validity of in‐depth, fine‐grained enquiry.^27^ Further recruitment from pledges made outside of this time was not necessary for this qualitative, exploratory study as data saturation was reached and no new themes or data became apparent in the final interviews.^28^ The mean interview duration was 48 min (with a range of 20–74 min). Table 1 provides information on the participants' characteristics, pledge and campaign exposure.

**Table 1:** Interview participant characteristics

	*n*
Members of the public	
Adults	10
Gender	
Male	6
Female	4
Ethnicity	
White	9
Black, African, Caribbean or Black British	1
Pledge recall	1
Selected pledge	
For infections that our bodies are good at fighting off on their own, like coughs, colds, sore throats and flu, I pledge to try treating the symptoms for 5 days rather than going to the GP	4
If I'm prescribed antibiotics, I will take them exactly as prescribed and never share them with others	3
If the NHS offers me a flu vaccination, I pledge to accept	1
It is vital we prevent antibiotics from getting into the environment. I pledge to always take any unused antibiotics to my pharmacy for safe disposal	1
[Created own pledge] To only seek to have antibiotics prescribed as a last resort, to reduce over use and the risk of increased antibiotic resistance.	1
Selected response to ‘How did you hear about us?’	
NHS	4
Community pharmacy	2
News Media	1
Colleague	1
Internet search	1
Family	1

The semistructured interviews were directed towards core topics of pledging to the campaign, prescribing, antibiotic use and propagating campaign awareness. This resulted in themes of campaign awareness, motivators to pledge (uncertainty about the future of ABR, personal gratification, personal responsibility, moral obligation and COVID‐19), perceptions of personal responsibility and moral obligation in clinicians, the impact of the campaign and campaign promotion. For a full list of themes and corresponding subthemes, see Table 2. Participant perceptions could fall into more than one theme. For example, participants could be motivated to pledge due to both uncertainties surrounding the future of ABR and personal responsibility.

**Table 2:** Themes and subthemes identified through a thematic and framework analysis of the transcribed interview data

Theme	Subtheme	*n*
Campaign awareness	Incidental internet searching	3
	Pre‐existing scientific interest and endeavours	3
	Professional networks	2
	Social media	2
	Cannot remember	2
Motivation to pledge	Uncertainty about the future of ABR	6
	Personal gratification	7
	Personal responsibility and moral obligation	4
Perceptions of prescribing	Widespread overprescribing	9
	Clinician‐dependent prescribing values	5
	Age‐related prescribing	6
Impact of campaign	Solidified existing beliefs	10
	Increased commitment to minimize ABR	6
	Increased antimicrobial resistance knowledge	3
Impact of COVID‐19	COVID‐19 irrelevant to pre‐existing beliefs	5
	Appreciation for infection prevention	3
	ABR deprioritized	3
Campaign promotion	Promotion to friends and family	5
	Antimicrobial resistance Stigma	5
	Wider targeting	4
	Frequency of messaging	9

*Note*: ‘*n*’ represents the participant frequency of theme occurrence.

Abbreviation: ABR, antibiotic resistance.

### 3.1 Campaign awareness

The majority of the group initially struggled to remember exactly where they became aware of the campaign but suggested a number of possible options, including through work, social media, friends and through scientific or academic groups they were already involved with. Routes of campaign exposure included World Antibiotic Awareness Week initiatives in the workplace that offered competition entry with AG sign‐up, routine emails, COVID‐19‐related Google searching and encouragement from friends who were AGs themselves.

### 3.2 Uncertainty about the future of ABR as a motivator to pledge

Following initial campaign exposure, participants were probed for perceptions that triggered a subsequent pledge and commitment to ABR. Motivations that stemmed from a fear of the future consequences of ABR were common.

**Participant 8**

We still need that sort of comfort blanket to fall back on, to know that if we need the Doctors, then the antibiotics are going to work.

**Participant 5**

It could happen so quickly. You won't have a choice, you can't backpedal.

This reflected a possible relationship between pledging and fear centred on the uncertainty of a future without antibiotics and the inability to reverse this fate should it manifest. Additionally, participants would often translate their fear of the consequences of ABR to their own personal lives and the needs of their loved ones. When talking about a family member, Participant 4 noted:

**Participant 4**

She seems to get a lot of antibiotics for nothing and it really concerns me, and I wanted a bit more of an insight into antibiotics and what they do and if there would be an issue for her later on in life.

Participant 4 shared a fear that if antibiotics were required for that family member in the future, they would not be effective due to historic excess use. Beyond fear of ABR, one individual also noted a fear of the cost of care in ABR, on personal and national levels, as a motivating factor that drove a desire to become involved in PHE's efforts. This shows a concern that supersedes the consequences of ineffective treatment in bacterial infections and spans broader to the eventualities of needing other support from the NHS if it becomes stringently strained for resources.

However, personal concern for ABR was not noted universally across the group and was not a predominant ‘general’ motivator to action. Additionally, there were indicators that the threat of ABR and statistics that may attempt to trigger an emotional response in fact deliver indifference. Participant 6 talked about hearing the figure that 10 million lives could be lost each year by 2050.

**Participant 6**

Is it actually too big, you know, to actually comprehend and how do you make that real? … We've become numb to the numbers … Sometimes people equate it [the death rate] to the equivalent of plane crashes and it's like ‘crikey, we wouldn't stand for that’. Yet, we accept this.

This suggests a potential disassociation from the data in the public realm and a numbness to the numbers when they are not tangible or perceived on a personal level.

### 3.3 Personal gratification as a motivator to pledge

A perception of personal gratification derived from a desire to champion positive behaviours was a second key motivator that drove the participants' antibiotic‐related behaviours and choose to involve themselves in the AG campaign. Many of the participants reported a personal gratification associated with being part of a larger collective action that transcended their own day‐to‐day antibiotic‐related behaviours.

**Participant 1**

It gave me that feeling that, you know, I was doing something good and being part of something that was big.

This demonstrates the influence of perceived gratification on perceptions. Altruistic intentions acted as an implied reason for engaging in judicious behaviour among AGs with a perceived fulfilment of these intentions achieved by pledging. By extension, this suggests that AGs' altruistic antibiotic‐related behaviours and the choice to pledge go together with solidarity and the concept that people depend on one another. Personal gratification also arose from personal interest.

**Participant 3**

I've been following issues on antimicrobial resistance … So, getting interested and getting to know more about how I can provide my help to prevent antimicrobial resistance.

This suggests that personal gratification may be derived from personal interest and the perception they were helping others. AGs' scientific interest in an area of public concern and a desire for driving action seemed to underpin campaign‐related perceptions in the cohort.

Participant 6 suggested that observed public ignorance stems from publications with mixed messaging and frequently changing perceptions towards health threats in the media.

**Participant 6**

This week it's that, now next week they'll have changed their minds and it'll be something else. Last week it was bad and this week it's alright.

Thus, a lack of commitment may stem from mixed messaging.

### 3.4 Personal responsibility and moral obligation as a motivator to pledge

Personal responsibility and moral obligation along with a perception of frustration towards others who did not share this sense of responsibility were also common.

A moral obligation in the context of personal responsibility to others/society was raised as a key motivator when pledging and, more generally, when following responsible antibiotic‐related beliefs. As a comparison to ABR responsibility, Participant 10 discussed a moral obligation to get the flu jab to avoid contracting or spreading the virus. This implies a perceived duty to prevent others from becoming ill and requiring health service support.

**Participant 2**

As someone who is advocating an end to antimicrobial resistance, therefore, it is upon me to act as an example.

A further participant in the 71–80 age group (Participant 7) expanded on this and suggested that their disinclination to visit the doctor unnecessarily arose from their childhood, implying that those who overuse doctor services and antibiotics are a less responsible sort of person:

**Participant 7**

I've always been disinclined to go to the doctors unless I really needed to. I think that stems from as a child, we had private doctors so we had to pay for them. And you didn't go for just trivial, silly things and I'm always irritated by people who do.

Others theorized that the perceived lack of knowledge and appreciation for antibiotic use among other members of the public is because it is assumed that some other party would take responsibility.

**Participant 6**

Somebody else can deal with that one … they'll do it for us.

Such views were observed with verbalized frustration and anger. Overall, this indicated a moral principle that bound the participant's actions to their perceptions of antibiotic use.

### 3.5 Perceptions of personal responsibility and moral obligation in clinicians

The perceived overprescribing of antibiotics by prescribers as a driver of ABR was an opinion reported by almost all participants. This too was linked to personal responsibility and moral obligation, but an obligation relative to the clinicians providing antibiotics. However, within this concept, viewpoints varied considerably, from perceptions that irresponsible overprescribing was common to some who perceived that overprescribing occurred, but it depended largely on the clinician, to others who felt overprescribing was rare and only occurred in cases of unavoidable patient pressures. The concept of pervasive antibiotic distribution was described as follows by Participant 1:

**Participant 1**

I think they're giving them out just sort of willy‐nilly … I wish they'd go and take the pledge as well and just say that they're not gonna give them out, sort of, you know, like Smarties. Because a lot of them do.

The time constraints of the consultation along with the influence of the patient on the GP were also acknowledged as influential factors, with Participant 9 noting:

**Participant 9**

Even if the GP is reluctant to prescribe, you know, they can be put under lots of pressure by patients … GPs, you know, they have seven minutes per patient.

The more general perception that the health service comprises both responsible and irresponsible prescribers was also common, and participants reported that the likelihood of antibiotic distribution depended on the individual clinician.

**Participant 9**

I think that a hundred percent it boils down to whom it is in question.

**Participant 10**

I was prescribed amoxicillin like smarties when I had recurring tonsillitis as a teenager, to the point where amoxicillin became totally useless on me.

Participant 10 went on to describe how, following recurrent tonsillitis, they had their tonsils removed through a private health service, a surgery not permitted through NHS services. Consequently, the procedure terminated any further need for tonsillitis‐related amoxicillin and emphasizes the role of other avenues of preventative medicine.

### 3.6 Impact of the campaign

Although there were subtle changes to the strength of AGs' motivations postpledge, in all cases, the participants reported pre‐existing perceptions and suggested that involvement in the campaign reinforced these perceptions:

**Participant 1**

I've always been one for not having antibiotics if I can help it.

**Participant 6**

I'd already been convinced to know what antibiotics are for.

Many of the participants had completed previous publicly accessible online courses on antimicrobial resistance, watched online presentations and been part of patient feedback groups. These actions are potentially the root of Participant 2's and Participant 5's comments:

**Participant 2**

It's a subject I related to, I understood the pros and cons.

**Participant 5**

It [pledging] was a bit easy for me to do actually.

When questioned about their perceived level of antimicrobial resistance knowledge, responses varied, with seven participants reporting that their knowledge had remained the same following a pledge. This was largely attributed to pre‐existing knowledge of antimicrobial resistance received through professional friends, extracurricular organization involvement or personal research related to their interest in the issue.

However, three of the participants reported increasing their knowledge and moral obligation to commit to tackling antimicrobial resistance following the pledge, reinforcing the idea that pledging is linked to personal gratification, responsibility and moral obligation:

**Participant 1**

Having actually signed the pledge and knowing that there is an organisation that's involved with that side of things, its made me want to, sort of, want to look into it more and get involved more.

**Participant 9**

I definitely did [increase my knowledge] at the time, when it was first mentioned, I definitely went and had a little Google.

### 3.7 Impact of COVID‐19 on motivation to pledge

Half of the cohort reported that the pandemic had not influenced their perceptions of ABR, the AG campaign or antibiotic use. Comparatively, in three cases, COVID‐19, including the associated restrictions such as lockdown, appeared to increase participants' appreciation of the ABR issue and AG campaign, as shown in the two examples below:

**Participant 1**

I was put on furlough when it first started, I'd got a lot more time on my hands anyway. So, I used that time to sort of go on the internet a lot more … Before I went on the internet, you know, I never knew about it at all. So, perhaps if COVID hadn't happened, perhaps I still wouldn't.

**Participant 5**

One of the upsides of the pandemic is that people are more focused on infection prevention.

Participant 1's comments highlight how the pandemic beneficially allowed them to actively come into contact with the AG campaign.

In direct contrast to an increased appreciation for infection prevention and ABR were opinions that ABR is a quieter message than it was before COVID‐19 and less important as a result, as noted by three AGs. One participant suggested that COVID‐19 had deprioritized the issue in the public's eye:

**Participant 2**

[ABR] is a smaller argument now, so, you know, it's like it's been replaced with ‘you need the vaccination’.

Likewise, it was perceived that COVID‐19 may have exacerbated the overprescribing of antibiotics that some participants attributed to patient pressures:

**Participant 2**

More antibiotics being used because people [prescribers] are panicking, ‘gosh I don't know what it is but I'll give them antibiotics’. So, sort of making the resistance situation worse.

This indicates a view that prescribers' commitments to weighing risk with nonmaleficence have become sensitized to the added risk of COVID‐19.

### 3.8 Campaign promotion

All of the cohorts reported discussing antibiotic‐related behaviours and perceptions with friends, family or co‐workers and five AGs had recommended the campaign to others. Participant 1 disclosed discussing the campaign with their spouse, while Participant 9 discussed guiding others along a thought process if it was raised. However, Participant 2 felt that this was equally true of misinformation:

**Participant 2**

People say ‘my Mums always sworn by antibiotics for everything’ and you're like, ‘hmm, ok’.

This suggests the contrasting challenge of social networks in ABR‐related perceptions. Other AGs, when asked if they promoted the campaign to others, replied that they had not due to not remembering their pledge to the campaign:

**Participant 7**

Until I got your email, I had totally forgot about it [the campaign].

Thus, this potentially indicates the need for more regular messaging in campaign promotion.

When touching on campaign promotion, the concept of ABR pledge stigma and disapproval of those who discussed ABR became apparent. This was evidenced by the comments below as AGs deliberated the prospect of discussing ABR and the AG campaign:

**Participant 1**

I sort of drop it into the conversations that I'm having with people and do it subtly so I don't sort of, people don't think ‘Oh my God, here he comes again, it's the antibiotic man’. I don't want that impression of me!

3.8.1

**Participant 5**

I would, yeah, risk being unpopular

**Participant 7**

It depends on the situation obviously, you don't want to lose too many friends over these things.

Thus, three AGs considered their views and the notion of discussing the topic as controversial. This indicates an experienced or anticipated rebuttal of opinions if they were to discuss antibiotic‐related consumption and disapproval for doing so.

When discussing the promotion of the campaign, the majority of participants communicated a desire for the AG campaign and other similar efforts to be communicated more widely and for contact to be more frequent:

**Participant 4**

I don't see much about it to be honest … People aren't coming to me to warn me about it.

This suggests a need and demand for broader targeting strategies. Contrastingly, Participant 7 reported that there is possibly too much messaging in the public realm:

**Participant 7**

You become saturated or people go ‘this is too much’ then shut off.

This raises the potentially challenging notion that there is already too much ‘noise’ in health messaging for campaigns to be heard and that the bombardment of the sheer volume of messaging in health can generate disengagement.

## 4 DISCUSSION

### 4.1 Campaign awareness and promotion

The public became aware of the AG campaign through various routes, emphasizing the broad, cross‐community campaign exposure that has been reported in earlier research.^15^ Most participants struggled to remember exactly where or how they encountered the campaign but provided various avenues of potential exposure, which reflected the actual varied routes of the exposure recorded in the participants' pledge data. Social media is undoubtedly a key part of this, with around one‐third of initial contacts made this way.^23^ Consequently, in the wider field of public health, this evidence highlights the importance of widespread marketing across communities to allow campaign dissemination to feed public commitment to ABR. The current study also supports the presence of self‐motivated individuals joining the campaign and making varied pledges, as opposed to top‐down interventions.

Likewise, most AGs could not recall their pledge, a finding similarly found in previous interview data,^15^ but in contrast to existing quantitative research.^29^ Kesten et al.^15^ attributed the difference in findings to the discrepancies in time between the individual's pledge and the time of research, with the researchers examining AGs 10–16 months postpledge compared to 5 months in the quantitative evaluation.^15^, ^29^ However, in this research, AGs were interviewed 7–8 months postpledge and the majority could still not recall their pledge. Being unable to remember one's pledge may potentially be because becoming an AG was not usually a commitment to new responsible behaviours but a solidifying of existing approaches, thus reducing the memorability of a specific pledged commitment. The significance of this finding to the current body of literature indicates that the act of pledging itself appeared to solidify beliefs in ABR, rather than the details surrounding the pledge. This is an important factor to note in future campaign development and further research could investigate whether this phenomenon is dependent on pre‐existing beliefs or reproducible in non‐AG cohorts.

#### 4.1.1 Pledge motivators

Pledging in the AG campaign appeared to foster the solidification of responsible perceptions that predated the individual's involvement in the campaign. However, there were also indicators that pledging acted as a trigger that encouraged participants to increase their knowledge of ABR. This reflects the results of previous AG knowledge research, which showed that ABR knowledge is greater in public AGs than in the EU Eurobarometer survey.^30^ This suggests that AGs hold a place as a valuable source of knowledge within the public, which has so far been untapped as part of the current campaign.

Furthermore, fears regarding their own health, and that of others, acted as a motivating catalyst for pledging to the AG campaign. It is perhaps unsurprising that some people taking time to visit the campaign website or pledge support, had some notion of the consequences of a world with increasingly ineffective antibiotics. Our findings suggest that individual members of the public are sufficiently concerned about ABR and this is an important feature to incorporate in ongoing and future health promotion interventions. Yet, using fear to promote healthy behaviours is historically controversial and has not been associated with desired outcomes in ABR.^31^ Thus, national and international campaigns could utilize this study's evidence for personal gratification as a motivator in ABR commitment and prudently utilize the Wellcome Trust's endorsement of a more open, upfront account of ABR, which makes it clear that individuals can take ‘immediate action’ to a ‘solvable’ problem.^32^

Similar to previous qualitative work, this study reinforces the significant role that perception of personal responsibility plays in motivating positive direction.^15^ The notion of altruistic intentions in personal responsibility was raised and is distinguished from egoistic intentions to use antibiotics judiciously. However, having interviewed just the public in this study, it is worth noting that a personal responsibility to address the ABR issue is not isolated to either healthcare professionals or the public but is instead a universal motivator in this pledge‐based campaign. Furthermore, although the AG campaign pledge system is positive for triggering a commitment and interest in ABR, drip‐feed messaging in the current AG and future campaigns are recommended to maintain engagement among the public, who, unlike health professionals, may not be exposed to the issue regularly.

### 4.2 Perceptions of prescribing

Some AG perceptions indicated that their clinicians' reasoning for prescribing antibiotics was not always congruent with their own, whether their perceptions were true or not. Previous research on the AG Campaign found the public was more likely to exhibit behaviours that reflected their pledge than healthcare professionals.^29^ From the perspective of members of the public, these amalgamated viewpoints stress that an AG status is one immersed in frustration towards the actions of others and unshared principles. This emphasizes the need for joint decision‐making and clear dialogue between prescribers and patients. However, perceptions of prescribing were highly varied; it was common for participants themselves to report variance in prescribing practices.

### 4.3 Impact of COVID‐19

Participants verbalized two somewhat contradictory perceptions towards COVID‐19, the first being a heightened appreciation for infection prevention and the second a deprioritizing of ABR in the face of a larger public health threat. The risk of COVID‐19 in the United Kingdom, as measured by death and infection rates, was significantly higher at the participants' point of pledge (November 2020) compared to the time of interview (June–July 2021).^33^, ^34^ Legal limits on social interactions also differed between the national lockdown through much of November 2020 and the staged relaxation of limits through the summer of 2021. Thus, it is theorized that views may have been influenced by public freedoms as well as the perceived threat of the virus as a by‐product of the case and death rate at the time, epidemiological spread and media‐conveyed data.^35^

### 4.4 Strengths and limitations

It is thought that the integrity and interparticipant dependability of this research was encouraged through a consistent approach to semistructured questioning and written communications.^36^ This study allowed the researcher to compare the perceptions and experiences of AGs who are nonhealthcare professionals, allowing for a comprehensive analysis of perceptions towards antibiotic use, the AG campaign and illness.^37^ Additionally, this study has provided insight into the real‐life experiences of AGs as members of the public and patients, an outlook that has not been formally captured in isolation.

Comparatively, the discrepancy between participants who activated their camera (seven participants) versus those who did not (three participants) may have led to inhomogeneity in the participant pool, given that the face‐to‐face element of interviews comes with the advantage of social cues that can guide the discussion.^38^ A relatively small sample size was used, a reflection of the eligibility criteria employed, to gain a snapshot of perspectives and data saturation was reached. The research may have inevitably been affected by recall bias and social desirability bias; participants may have been more likely to remember instances of positive antibiotic‐related perceptions and behaviours given the nature of the research.^39^ Similarly, participants were recruited on a volunteer basis and therefore this research may have been affected by volunteer bias, one form of selection bias.^40^ This is particularly so as AGs who had a previous interest in the subject may have been more likely to pledge as an AG and volunteer for the study.

## 5 CONCLUSION

For the first time, this research has provided direct insight into the reasons why members of the public pledge to support a continuing AG campaign. People pledging were clearly motivated to continue optimizing antibiotic use as a way of containing resistance, at times driven by their own fears of the consequences if they and others failed to take action. In many cases, pledging solidified existing values and behaviours, while in other cases, this study found personal gratification to be important to inspiring action. Resultantly, improved collaboration and more frequent messaging between leading campaign makers and the public will be vital to acknowledge the relationship between ABR development, social barriers, antimicrobial prescribing and human health.

## AUTHOR CONTRIBUTIONS

Lorna Flintham carried out the study, developed the main conceptual ideas and led the data analysis. Lorna Flintham, Diane Ashiru‐Oredope and Elizabeth Dalgarno conceived and designed the study. Elizabeth Dalgarno developed the theory, supported the analysis and interpretation of results, provided guidance in all study aspects and supervised Lorna Flintham throughout the project. Diane Ashiru‐Oredope and Jordan Charlesworth contributed to the methodology and Jordan Charlesworth implemented the participant eligibility criteria, and population targeting and led initial participant communications. R.H. and Diane Ashiru‐Oredope devised the project and facilitated interorganization collaboration and communications. Roger Harrison had considerable input into the design and content of the paper, working with all other authors as part of an academic team. This included approving the results and their wider implications. Lorna Flintham, Elizabeth Dalgarno, Diane Ashiru‐Oredope, Jordan Charlesworth and Roger Harrison contributed to the interpretation of the results. Lorna Flintham wrote the manuscript with input from all authors. All authors provided critical feedback and helped shape the research, analysis and manuscript.

## 6 ETHICS STATEMENT

Ethical permission for the research was gained from The University of Manchester's University Research Ethics Committee (UREC), which granted proportionate UREC approval (reference number 2021‐11584‐19056). Written consent was gained from all participants before the interview.

## Data Availability

The anonymized data sets, free from personal identifiers, generated during and/or analysed during the current study are available from the corresponding author on reasonable request.
